# A personalized and evolutionary algorithm for interpretable EEG epilepsy seizure prediction

**DOI:** 10.1038/s41598-021-82828-7

**Published:** 2021-02-09

**Authors:** Mauro. F. Pinto, Adriana Leal, Fábio Lopes, António Dourado, Pedro Martins, César A. Teixeira

**Affiliations:** Univ Coimbra, Centre for Informatics and Systems of the University of Coimbra, Department of Informatics Engineering, Coimbra, Portugal

**Keywords:** Machine learning, Predictive medicine, Epilepsy

## Abstract

Seizure prediction may improve the quality of life of patients suffering from drug-resistant epilepsy, which accounts for about 30% of the total epileptic patients. The pre-ictal period determination, characterized by a transitional stage between normal brain activity and seizure, is a critical step. Past approaches failed to attain real-world applicability due to lack of generalization capacity. More recently, deep learning techniques may outperform traditional classifiers and handle time dependencies. However, despite the existing efforts for providing interpretable insights, clinicians may not be willing to make high-stake decisions based on them. Furthermore, a disadvantageous aspect of the more usual seizure prediction pipeline is its modularity and significant independence between stages. An alternative could be the construction of a search algorithm that, while considering pipeline stages’ synergy, fine-tunes the selection of a reduced set of features that are widely used in the literature and computationally efficient. With extracranial recordings from 19 patients suffering from temporal-lobe seizures, we developed a patient-specific evolutionary optimization strategy, aiming to generate the optimal set of features for seizure prediction with a logistic regression classifier, which was tested prospectively in a total of 49 seizures and 710 h of continuous recording and performed above chance for 32% of patients, using a surrogate predictor. These results demonstrate the hypothesis of pre-ictal period identification without the loss of interpretability, which may help understanding brain dynamics leading to seizures and improve prediction algorithms.

## Introduction

Epilepsy is a brain chronic disorder affecting people of all ages and conditions. With approximately 1% of the world population suffering from this condition, it is one of the most common neurological diseases^1^. Besides its social impact related to discrimination and stigma, it is associated with a significant economic impact regarding health care needs, premature death, and loss of productivity. While seizure control may be achieved with a success rate of 70% by delivering antiepileptic drugs^2,3^, Drug-Resistant Epilepsy (DRE) patients require strategies, such as seizure prediction, to improve their lives^4–6^.

The main goal of seizure prediction is to anticipate a seizure by timely raising an alarm. The existence of an efficient seizure prediction algorithm may, in the first place, open the way to seizure-suppression medication or to the development of closed-loop systems that, automatically, trigger some seizure disarming procedure. In the second place, it may also minimize subsequent effects from it, such as anxiety and social exposition. The selection of adequate parameters for seizure prediction models must consider all performance indicators simultaneously, such as sensitivity, false prediction rate per hour (FPR/h), seizure occurrence period (SOP), and seizure prediction horizon (SPH). SPH is the minimum interval, between an alarm and a seizure that renders an intervention possible. The time frame where the seizure is predicted to occur is named SOP^7,8^.

The interest in Electroencephalogram (EEG)-based seizure prediction algorithms started in the 1970s and has gradually increased. This signal is used to continuously monitor epileptic patients and has proved to be useful for pre-surgical evaluation and diagnosis of DRE patients^4,9^. It is possible to segment an epileptic patient’s EEG in four periods: pre-ictal, which precedes the seizure; ictal, corresponding to the seizure; post-ictal, which follows the seizure; and finally, inter-ictal, which is a seizure-free time frame that can be found in between the post-ictal and the pre-ictal of consecutive seizures. The most difficult period to be detected is the pre-ictal, as it is not clinically annotated and a recurrent pattern was not detected so far^10–12^. Moreover, studies^13,14^ have proved that this period is associated with significant inter- and intra-patient heterogeneity. Besides, the presence of confounding factors^10,15^ as alterations in brain dynamics due to circadian effects, medication, stress situations, and others, can induce significant changes in features distribution. There is also a high-class imbalance: the inter-ictal period is extremely long when compared to the pre-ictal one^16^.

In recent years, the European Epilepsy Database^1,4^ was developed in the context of the European EPILEPSIAE project (www.epilepsiae.eu), where several studies^17–19^ reported that in realistic scenarios, seizure prediction above chance level was only possible for a very small number of patients (10%). Some studies^17,18,20^ considered a standard framework that consists of preprocessing, feature extraction, feature selection, classification, output regularization, and performance evaluation, where pre-ictal period determination and feature extraction tended to be the most critical decisions. Authors typically define a pre-ictal time and split the EEG into individual and independent windows of fixed size, labelling each as inter-ictal, pre-ictal, ictal, and post-ictal. Usually, the choice of a fixed pre-ictal period follows a grid search approach of different periods, e.g., 2, 20, 30, 60, or even 240 min^21,22^. This pipeline has two limitations. Firstly, feature selection is commonly based on the discriminating power of each feature individually, or by using wrappers and embedded methods that address synergies but require a large computational power^14,16^. Secondly, this framework is modular and composed of independent stages, where feature selection is usually not based in the final seizure prediction performance but rather in distinguishing pre-ictal from inter-ictal independent windows of fixed size. Therefore, the interaction between stages is not handled. Additionally, a fixed sub-set of electrodes and features are often considered at a given time instant, not allowing for the evaluation of lagged values of corresponding features, i.e., not considering temporal dynamics. More recently, Deep Learning models, such as Recurrent Neural Networks (RNNs), Long Short Term Memory (LSTM) and Bi-LSTM, were introduced in seizure prediction^23,24^. Due to their underlying mechanisms, they are more suitable for time-series analysis than traditional classifiers. Despite the theoretical potential of these models to handle brain dynamics and the existence of notable efforts to retrieve interpretable insights (where the EEG signal is no exception^25,26^), clinicians may not be willing to make high-stake decisions based on them^27^. Low-complexity algorithms with interpretable insights (as the ones using intrinsically interpretable models), able to provide a deeper understanding of the ictogenesis process, should be favoured over others^10,11,28^, because they enable analysis by clinicians, and consequently improves confidence on the given performance.

To tackle the aspects of interpretability, synergy concerning features, and interaction between all pipeline stages, a solution may lie in the construction of a search algorithm that selects a reduced set of computationally efficient and widely used features. This search algorithm should select features by looking at the pipeline as a whole, and not as a sequence of independent stages. We propose an Evolutionary Algorithm (EA) to handle this problem, as these type of algorithms have become effective for several tasks such as direct search, optimization and machine learning problems^29^. They can be seen as population-based search algorithms that mimic natural evolution by evaluating the quality of individuals through the use of evolution operators (crossover and mutation) and a fitness function. A population is a group of individuals, where each one is represented by a point in the search space. The fittest individuals, evaluated by their fitness function values, tend to survive and propagate the genetic material by reproducing or mutating^29–31^.

We developed a patient-specific search algorithm aiming at seizure prediction while trying to discover the best pre-ictal time, based on evolutionary computation, where each individual in the EA population is a set of five features. Simply put, the set of features (individuals) that best perform in seizure prediction using a logistic regression classifier (fitness function), survive and proliferate, while the remaining die and do not contribute to propagate their genes, similarly to natural selection. This method, besides using the predictive power of a set of features and their synergy, tries to provide a deeper understanding on the seizure generation processes by considering a sequence of instants instead of analyzing only one instant, and by giving results that can be interpretable. In the end, we demonstrate the interpretability of the EA output and how to extract patient-specific knowledge from it.

## Materials and methods

**Figure 1:**
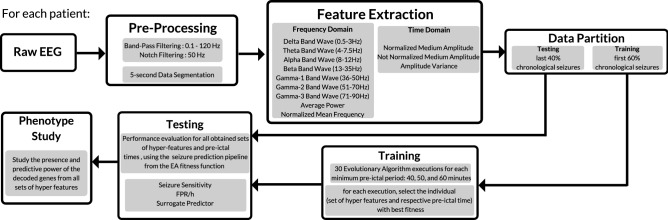
Flowchart of the proposed work for each patient, comprising data processing, feature extraction, training, testing, performance evaluation and phenotype study.

The strategy followed can be divided into data preprocessing, feature extraction, training, testing, and phenotype study (see Fig. 1). In short, the raw EEG is filtered and segmented into time-windows from which features are extracted. Then, the first 60% chronological seizures are used as input to the EA, which is executed 30 times. The best individual (set of features and correspondent pre-ictal time) of each execution is selected. The EA output features are then tested and evaluated with the last 40% chronological seizures. An SPH of 10 min was used both in training and testing stages. This procedure is explored for three different minimum pre-ictal periods: 40, 50, and 60 min. If the results for a given patient are satisfactory, a phenotype study can be made. These steps are described in this section and are shown for one patient, as our approach is patient-specific.

Regarding the EA output features, these will be based on a feature construction^32–34^ process: the application of a set of constructive operators to a set of existing features, which results in the construction of new ones. The latter are believed to be more powerful, as these higher-level generated features take into account the interactions in the previous feature space. In this case, the features from the feature extraction stage (first-level features) will be used to construct second-level ones by windowing and applying a mathematical operator, constituting the phenotype features. In the following, first-level features will be named as *features*, while the second-level ones will be referred to as *hyper-features*.

### Database

From the European Epilepsy Database also known as EPILEPSIAE database^1,4^, 19 DRE patients (11 males and 8 females, aged 40.26±13.52 years) from the Universitätsklinikum Freiburg in Germany were selected. The dataset comprises 120 seizures (71 for training and 49 for testing), 284 h of training data and 710 h of testing data ($\approx$ 1 month). It is important to mention that, for training seizures, we selected only the last recorded 4 h before each seizure. Nevertheless, our testing data is continuous, as it comprises all available inter-ictal data without any segment removal. Our patient selection criteria were the following: (i) patients containing only seizures with focus on the temporal lobe, as these are the most representative in DRE patients; (ii) patients having an average of 2–5 daily seizures and a minimum of 5 recorded seizures that were separated by periods of at least 4 h; and (iii) EEG scalp recorded specifically with a sampling frequency of 256 Hz. We selected only patients with temporal lobe epilepsy as it is the most common type of focal epilepsies^35^. All electrodes were placed according to the 10–20 system. The data was collected while patients were in the clinic for routine pre-surgical monitoring. The use of this data for research proposals has been approved by the Ethical Committee of the three hospitals involved on the database development (Ethik-Kommission der Albert-Ludwigs-Universität, Freiburg; Comité consultatif sur le traitement de l’information en matière de recherche dans le domaine de la santé, Pitié-Salpêtrière University Hospital; and Ethics Committee of the Coimbra University Hospital). All methods were performed following the relevant guidelines and regulations. Informed written patient consent was also obtained.

### Pre-processing and feature extraction

The data was filtered with a 50 Hz notch filter and with a 0.1–120 Hz bandpass filter. Then, a 5-s non-overlapping window was chosen to segment the recordings. For each time window in each electrode, the following features were extracted in the frequency domain: relative power in delta (0.5–3.5 Hz), theta (4–7.5 Hz), alpha (8–12 Hz), beta (13–35 Hz), and in three gamma sub-bands (36–50 Hz, 51–70 Hz and 71–90 Hz), average power, and mean normalized frequency. As the frequency limit of gamma activity is not consensual among authors, and its division into high-gamma and low-gamma is not uncommon^36^, we decided to divide it. The normalized and non-normalized (with respect to the maximum value in each window) mean amplitude, and the amplitude variance, were extracted as well.

### Evolutionary algorithm

**Figure 2:**
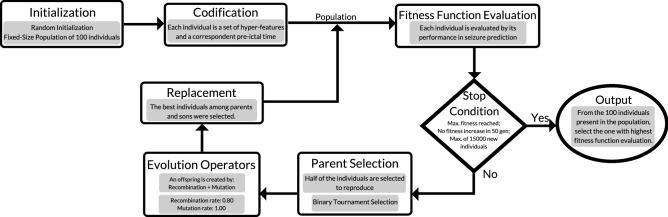
The proposed GA configuration.

We used a Genetic Algorithm (GA) as the EA, whose steps are described in Fig. 2. Population, which is a set of possible solutions, is initialized randomly with a fixed number of 100 individuals. Each individual has its encoding, which can be seen as the bridge between the problem context and the problem-solving space, where phenotypes (possible solutions) are encoded into genotypes (a chain of characters coded from the individual). Then, each individual is evaluated based on the fitness function, which is a mathematical criterion that results in a measure related to the seizure prediction performance. Then, half of the individuals (parents) are selected to reproduce through binary tournament selection (parent selection). The evolution operators are recombination and mutation, where we used a recombination rate of 0.80 (80% of times, two parents produced an offspring) and a mutation rate of 1.00 (all offspring suffered a mutation). The individuals among parents and offspring with the best fitness evaluation are selected and comprise the next generation of individuals ((1+$\lambda$) replacement strategy^29^). Evolution occurred until one of the following criteria was met: i) maximum fitness was reached, ii) fitness did not increase over the last 50 generations, iii) 15000 new individual evaluations were performed (see Supplementary Material for more information concerning the EA configuration). As a reduced computation time is desired in order to have real-life applicability, a fast convergence is desired. Thus, a greedy approach was used, i.e., points offering the most obvious and immediate benefits are chosen. Despite this strategy may not usually produce an optimal solution, it is believed that it approximates the global optimum one in a reasonable amount of time^37^.

#### Codification and evolution operators

**Figure 3:**
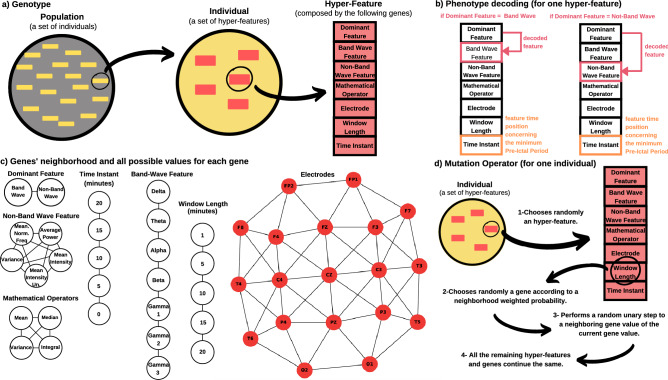
An illustrated scheme of genotype (**a**), phenotype decoding (**b**), gene’s neighbourhood and all possible values for each gene (**c**), and the mutation operator functioning (**d**).

Figure 3 illustrates the rationale behind genotype, phenotype decoding and mutation. Concerning genotype, a population is represented by a group of individuals, where each one is defined by five hyper-features. Each hyper-feature is encoded with seven genes: dominant feature, band-wave feature, non-band wave feature, mathematical operator, electrode, window length and time instant (minutes before the minimum pre-ictal period) (see Fig. 3a).

Genotype-phenotype mapping (see Fig. 3b) consists in: (i) finding the feature that will be decoded to the phenotype for each hyper-feature by inspecting the dominant feature gene, e.g. if the dominant feature gene value is band-wave, then the band-wave feature is decoded; (ii) constructing each hyper-feature by windowing the decoded feature, from the given electrode, within the window length, and then by applying the respective mathematical operator; and (iii) placing each hyper-feature chronologically in a timeline according to its respective time instant and obtaining the pre-ictal period (the temporal distance between the first chronological hyper-feature and the seizure). The latter allows to not only analyzing a sequence of instants instead of only one instant but also to find the best pre-ictal time (see Supplementary Material for a genotype-phenotype decoding example). Then, with the hyper-features constructed and placed chronologically, it is possible to perform sliding-window analysis, classification, regularization and evaluation, all these addressed in the fitness function.

In Fig. 3c, one can visualize all possible values for each gene and its neighbourhood that must be established to perform recombination and mutation. These neighbourhoods were designed while accounting the relationship between each gene value (see Supplementary Materials for more details). Mutation, interpreted as a unitary step that will cause a random and unbiased change^29^, occurs in the following form for an individual (see Fig. 3d): one of the hyper-features is chosen randomly, and then one gene of that hyper-feature is chosen randomly to mutate. The remaining hyper-features and genes continue unaltered. Recombination is a stochastic operator that combines genetic information from two parents (individuals) into one or more offspring^29^. After selecting two parents to reproduce, this operator performs the recombination of all paired hyper-features. Thus, hyper-feature pairing is the first step and then, the recombination operator works at the hyper-feature gene level. Each offspring gene value is obtained by choosing a random one belonging to the shortest path between the correspondent two parent gene values (see Supplementary Material for more details concerning evolution operators, and an example for each).

#### Fitness function

**Figure 4:**
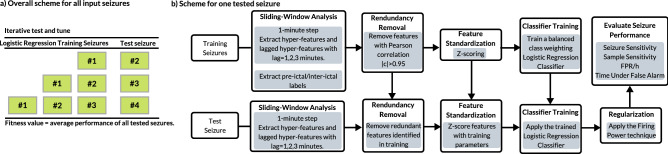
An illustrated scheme of the iterative retraining and testing evaluation (**a**) and the used prediction procedure for one tested seizure (**b**).

An individual fitness evaluation is made iteratively (retraining the logistic regression classifier with new seizures) according to seizure prediction performance (see Fig. 4a). For each tested seizure (see Fig. 4b), hyper-features and labels are collected from previous seizures by performing time-moving analysis (1-min step). Delayed features with a lag $l=1,2,3$ min are also extracted, which has the objective of transforming static hyper-features into temporal ones since the used classifier does not handle time explicitly. Redundancy is handled, by removing features with an absolute correlation coefficient $\left| \rho \right| >0.95$. Then, they are standardized by a *z*-score process.

Before classifier training, classes’ weight was balanced with an inverse proportion to their frequency of occurrence (see Supplementary Material for mathematical formulation). We chose the logistic regression classifier, as it is computationally light, its decision curve takes the form of a logistic function, it is an intrinsically interpretable model and therefore, incorporates interpretability directly to its structure^27,38,39^. It models the probability $p(\mathbf{x })$ of a sample $\mathbf{x }$ with *n* predictors belonging to a certain class as shown in Eq. (1)^40^, where $\beta _{n}$ is the regression coefficient value concerning the hyper-feature $x_{n}$:

1 $$\begin{aligned} p(\mathbf{x })= \frac{1}{1+e^{-(\beta _{0}+\beta _{1}x_{1}+\beta _{2}x_{2}...+ \beta _{n}x_{n})}}. \end{aligned}$$

For the tested seizure, the same procedure was applied, but using *z*-score parameters and logistic regression from training seizures. Then, a regularization technique is applied as it is desired to have a predictor robust to noise: the Firing Power^41^ (see Supplementary Material for mathematical formulation). It quantifies the classifier rate output as pre-ictal in a past-time window with the size of the pre-ictal period. When an arbitrary threshold (the maximum tolerance for the prediction error) is surpassed, an alarm is triggered. The latter was set to a reasonable limit of 0.70.

Prediction performance is based on four measures: seizure sensitivity $S_{p}$ (the ratio of correctly predicted seizures), sample sensitivity $S_{s}$ (ratio of samples classified as pre-ictal within all pre-ictal samples); time under false alarm $T_{f}$ (ratio of samples classified as pre-ictal within all inter-ictal samples); and FPR/h (number of false alarms divided by the total time inter-ictal period).

Accordingly, the performance for a tested seizure is given by Eq. (2), which we consider optimal if its value is 1. The latter corresponds to correctly predicting all samples ($S_{s}$=1 and $T_{f}=0$) and therefore, predicting the seizure ($S_{p}=1$) while not triggering any false alarm (FPR/h=0). By measuring these metrics simultaneously, performance is not only based on a seizure prediction system, but also on correctly classifying the maximum number of samples. Furthermore, FPR/h is multiplied by $T_{f}$, as it is not meaningful to have the same $T_{f}$ with different number of alarms: it is preferred to have a shorter number of false alarms. Thus, it emphasizes the downside of having simultaneously a high FPR/h and a longer $T_{f}$. Finally, the fitness function evaluation is obtained by averaging all tested seizure performances:

2 $$\begin{aligned} Performance=(S_{s}+S_{p})\times 0.5 - FPR/h\times (1 + T_{f}). \end{aligned}$$

#### Training, testing and statistical validation

For a patient, in a real-life context, one would choose a determined minimum pre-ictal period and would run one execution of the EA. The best set of features would then be used for predicting seizures. In an academic context, as this paper concerns an exploratory study, we executed an EA 30 times for each patient, for each minimum pre-ictal period. These sets of selected features were then prospectively tested with the patient’s past 40% seizures (unseen data), using the same pipeline used for the fitness function but with one extra step: after raising an alarm, a refractory period of SOP+SPH was used. Due to the latter, we excluded refractory periods from the inter-ictal period in our FPR/h calculations, in order to only account for the period during which false alarms can be triggered, and which enables a proper comparison with other methods^16^. Performance is based on $S_{p}$, FPR/h and comparison with a surrogate predictor^42^. The latter was implemented with the objective of understanding if the proposed algorithm performed above chance level.

The surrogate predictor makes use of Monte Carlo simulations by random shifting seizure times. A model is considered to perform above chance if its performance is higher than the surrogate one with statistical significance, under the following null hypothesis that the proposed method performance is not above chance level. Unspecific methods, as the random predictor^43^, which assume that alarms are triggered randomly without using information from the EEG signal, are also commonly used. Nevertheless, despite a surrogate approach requires more computation time, it offers greater confidence in determining if a model performs above chance^11^ (see Supplementary Materials for a detailed implementation of our surrogate predictor).

Since we performed 30 executions for each minimum pre-ictal period, we did two different statistical validations: one on the overall set of executions and another on the number of executions that perform above chance. Concerning the first, we considered a performance to be above chance level if its average value is higher than the one observed for the averaged surrogate predictor, with statistical significance of $\alpha$=0.01 (using a one-tailed $t-test$). The second validation is related to the real-life context, where we would only run one execution (and not 30) of the EA and use it: we need to understand how likely the selected features would perform above chance level, and if that probability is statistically significant. Towards the latter, we calculated the number of executions that outperformed the surrogate predictor with statistical significance of $\alpha$=0.01 and verified if this number was significant for the whole set of 30 executions, by comparing the obtained ratio with the one from a binomial distribution (this procedure was inspired by Alvarado Rojas et al.^19^, as they used it to check if the number of validated patients was significant for the whole group). Thus, for a significance of $\alpha$=0.05, the probability to observe, for at least *i* of *I* (individuals) executions that outperformed the surrogate predictor, is given by:

3 $$\begin{aligned} P_{binom}(i,I,\alpha ) = \sum _{j=i}^{I}\left( {\begin{array}{c}I\\ j\end{array}}\right) \alpha ^{j}(1-\alpha )^{(I-j)} \end{aligned}$$

#### Phenotype study

As EAs are associated with random components (as initialization, parent selection, and evolution operators), it is possible to obtain, for each execution, a different solution (set of hyper-features) with similar performance. Thus, the objective of performing a phenotype study is to understand the overall influence of each gene value. It is possible to calculate the gene value predictive power from a gene using by assigning to it the absolute of the correspondent logistic regression coefficient. Presence was also studied, where a binary value (1/0) was assigned considering the gene value presence in a hyper-feature. By computing these values to all hyper-features that compose an individual, one obtains the correspondent gene value predictive power for an individual. After this, one can compute the correspondent normalized gene value predictive power and normalized presence for all individuals (see Supplementary Material for more details and for mathematical formulation).

## Results and discussion

**Figure 5:**
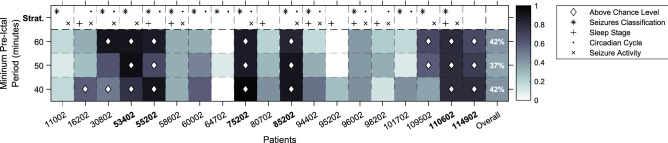
The performance of all patient-models, organized by the minimum pre-ictal period. Colour represents the ratio of executions that outperformed the surrogate predictor, while the diamond shape means that a given overall set of 30 executions outperformed the surrogate predictor. On the top line, patient stratification is presented concerning seizure classification (*, FOA or FOIA/FBTC seizures), sleep stage at seizure onset (+, sleep/wake), circadian cycle at seizure onset (., day/night) and annotated activity pattern (x, rhythmic/non-rhythmic). On the overall column, one can see the ratio of patients whose models overall performance is above chance level.

Figure 5 presents the statistical validation on testing seizures for all patients and for all minimum pre-ictal periods, along with patient stratification. Thus, colour represents the ratio of executions (*N* executions out of 30) that outperformed the surrogate predictor while the diamond-shaped marker represents which sets of executions had an overall performance above chance level. It is possible to see that, for 40, 50, and 60 min of minimum pre-ictal periods, 42% (8 in 19), 37% (7 in 19), and 42% (8 in 19) of patient models performed above chance level, respectively. Furthermore, 32% (6 in 19) presented a performance above chance level for all three pre-ictal periods, and therefore, we consider this value as our number of validated patients, and 48% (9 in 19) for at least one pre-ictal period. Furthermore, it was possible to develop a significant number of executions that are significant for the whole set, for 89% (17 in 19) of patient-models for all three pre-ictal periods simultaneously, and for 100% (19 in 19) for at least one pre-ictal period.

The average fitness value in training was $0.62\pm 0.12$ and the SOP duration was $40.46\pm 8.85$ min. In testing, we obtained an $S_{p}$ of $0.38\pm 0.19$, $0.36\pm 0.24$, and $0.37\pm 0.27$, and an FPR/h of $1.03\pm 0.84$, $0.76\pm 0.39$, and $0.58\pm 0.31$ for the minimum pre-ictal periods of 40, 50, and 60 min, respectively.

Patient stratification was based on seizure classification (FOA or FOIA/FBTC), seizure activity (rhythmic/non-rhythmic), sleep stages (sleep/awake), the period of the day (night/day, with 10 pm and 7 am as time thresholds^19^) at seizure onset. In these, a patient was selected if a determined stratification criterion was met both in training and testing seizures. Activity pattern was the only criterion that improved significantly the percentage of patient models performing above chance for at least one pre-ictal period: 58% (7 in 12). With the remaining stratification parameters, the obtained percentages were 50%, 42% and 33% for seizure classification, circadian cycle, and sleep stage, respectively. Concerning $S_{p}$, it is worth mentioning that we have obtained a Pearson correlation coefficient of $\rho =0.39$ and $\rho =0.29$ between this metric and stratification criteria of seizure classification and activity pattern, respectively.

Table 1 presents training and testing results, as well as information concerning the considered group of patients: number of seizures and recording duration, seizure activity pattern, seizure classification, and day period (i.e. Day or Night) at seizure onset. It is worth noting that we are only presenting here one set of 30 executions for each patient, which corresponds to the pre-ictal period that presented the best performance. One can find the performance for all patient-models in Table S2 from Supplementary Material. In fact, one could have included the pre-ictal period in the genotype instead of searching for different minimum values, but this can be justified by the fact that its duration influences directly the used seizure performance metrics. By including the pre-ictal period in the genotype, we would take the risk of seeing pre-ictal time changes just because it would immediately increase fitness value, while not being related to brain dynamics. Furthermore, EA hyper-features are also capable of increasing the pre-ictal period duration through the increase of all hyper-features’ time instant, as explained in genotype-phenotype mapping. This operation was allowed since it depends on all hyper-features simultaneously. Moreover, experimenting with different and consecutive pre-ictal periods allowed us to explore the idea of seizure susceptibility that may be envisioned as a regression problem^10^. The fact that it was possible to build prediction models that achieved performance above chance level for all tested three pre-ictal periods, for 32% of patients, might suggest this.

**Table 1:** Patient information and results for the EA 30 executions.

Patient ID	#Seizures (Training/ Testing)	Recording Duration (hours)	Seizure Activity Pattern	Seizure Classification	Onset Day Period	Sleep Stage	Training		Testing			
							SOP (minutes)	Fitness	Sensitivity ($S_{p}$)	FPR/h	Surrogate Sensitivity	Ratio of Executions Above Chance
11002	3 2	12 15.94	?, s, a t, t	UC, FOIA, FOIA FOIA, FOA	N, N, D D, D	A,R,A A, A	52.83±3.34	0.82±0.09	0.12±0.21	0.37±0.15	0.25±0.14	0.17*
16202	4 3	16 21.81	r, ?, r, r r, ?, r	UC, FBTC, UC, FOIA FOIA, FOIA, FOIA	N, N, N, D N, D, D	A, A, A, A A, A, A	31.00±2.00	0.58±0.09	0.30±0.26	0.49±0.33	0.16±0.09*	0.60*
30802	5 3	20 44.41	t, t, t, t, t t, t, t	FOA, FOA, FOA, FOA, FOA FOA, FOA, FOA	N, N, D, N, N N, D, N	R, A, 2, A, A R, 2, 2	50.83±2.61	0.46±0.08	0.70±0.20	0.55±0.47	0.40±0.12*	0.90*
**53402**	**3** **2**	**12** **50.75**	**?, ?, ?** **?, t**	**FOA, FOA, FOA** **FOA, FOIA**	**D, D, N** **D, D**	**A, A, 2** **A, A**	**43.83**±**2.79**	**0.84**±**0.08**	**0.78**±**0.31**	**0.35**±**0.11**	**0.22**±0.08*	0.93*
**55202**	**5** **3**	**20** **43.33**	**t, d, t, t, t** **t, r, r**	**FOIA, FOIA, FOA, UC, UC** **FOA, FUC, FOIA**	**D, D, D, D, D** **D, D, D**	**A, A, A, A, A** **A, A, A**	**31.83**±**2.41**	**0.59**±**0.04**	**0.70**±**0.22**	**1.21**±**0.42**	**0.36**±0.09*	0.90*
58602	4 3	16 15.19	r, t, t, r r, r, t	FOIA, FOIA, FOIA, FOIA FOIA, FOIA, FOIA	D, N, D, D D, D, N	A, R, A, A A, A, 2	51.67±2.69	0.48±0.11	0.16±0.17	0.25±0.27	0.12±0.11	0.33*
60002	4 2	16 46.61	d, c, t, t d, d	FOIA, FOIA, FOIA, UC FOIA, FOIA	N, N, D, N N, N	1, A, A, R R, 1	35.00±4.08	0.68±0.12	0.48±0.30	1.29±0.52	0.36±0.09	0.60*
64702	3 2	12 31.98	?, m, t t, t	FOA, FBTC, FBTC FBTC, FBTC	D, N, D D, N	A, A, A A, 2	44.17±3.67	0.71±0.14	0.05±0.15	0.73±0.39	0.18±0.05	0.10*
**75202**	**4** **3**	**12** **31.67**	**t, t, t, t** **t, ?, t**	**FOA, FOA, FOA UC** **FOA, FOA, FOA**	**N, N, D, D** **D, D, N**	**2, 2, A, A** **A, A, A**	**31.67**±**2.98**	**0.59**±**0.10**	**0.70**±**0.22**	**0.69**±**0.33**	**0.28**±0.07*	0.97*
80702	4 3	16 24.06	b, b, ?, c m, c, c	FOIA, FOIA, FOIA, UC UC, FBTC, FOIA	N, D, D, D D, D, D	A, A, A, A A, A, A	34.50±3.95	0.50±0.08	0.31±0.21	0.65±0.34	0.31±0.10	0.43*
**85202**	**3** **2**	**12** **20.71**	**m, c, m** **m, m**	**FOIA, FOIA, UC** **UC, UC**	**N, D, N** **D, N**	**2, A, A** **A, A**	**34.67**±**2.87**	**0.71**±**0.04**	**0.47**±**0.18**	**0.36**±**0.34**	**0.16**±0.08*	0.90*
94402	4 3	16 24.18	?, d, b, t ?, b, ?	FOA, UC, FOIA, UC FOA, UC, FOA	D, D, D, N D, N, D	A, A, A, 2 A, 2, A	36.17±2.11	0.63±0.06	0.38±0.24	1.05±0.58	0.31±0.13	0.53*
95202	4 3	16 77.46	b, b, b, m b, b, t	FBTC, FOIA, FOIA, FOIA UC, FOIA, UC	N, D, N, D N, N, N	2, 2, 2, 2 2, 2, 2	35.33±3.14	0.52±0.12	0.14±0.17	0.66±0.34	0.23±0.09	0.27*
96002	4 3	16 76.34	t, t, t, d a, t, a	FOIA, FOIA, FOIA, FOIA UC, FOIA, FOIA	D, D, D, N N, D, N	A, A, A, A A, A, A	30.50±1.98	0.52±0.12	0.23±0.20	0.77±0.53	0.24±0.10	0.43*
98202	4 3	16 47.47	t, a, t, t t, t, t	FOIA, FOIA, FOIA, FBTC FOIA, FOIA, UC	N, D, D, D N, D, D	A, A, A, A A, A, A	42.00±2.77	0.54±0.16	0.31±0.32	1.66±1.23	0.35±0.18	0.33*
101702	3 2	12 24.12	t, t, t r, r	FOIA, FOIA, FOIA FOIA, FOIA	D, D, D D, D	A, A, A 2, A	33.00±2.77	0.55±0.08	0.23±0.28	0.44±0.28	0.17±0.10	0.43*
109502	3 2	12 45.99	t, t, t, t, t	FOIA, FOIA, FOIA UC, UC	D, D, N D, D	A, A, 1 A, A	54.17±3.44	0.77±0.16	0.83±0.24	1.35±1.35	0.58±0.12*	0.67*
**110602**	**3** **2**	**12** **26.2**	**t, t, t** **t, t**	**FOIA, FOIA, FOIA** **FOIA, FOA**	**D, D, D** **N, D**	**A, A, A** **A, A**	**52.50**±**3.59**	**0.79**±**0.08**	**0.50**±**0.26**	**0.33**±**0.24**	**0.21**±0.11*	0.80*
**114902**	**4** **3**	**16** **41.35**	**s, b, s, t** **r, a, t**	**FOA, FOIA, FOIA, FBTC** **UC, FOIA, FOIA**	**D, D, D, N** **D, D, D**	**A, A, A, 2** **A, A, A**	**33.67**±**3.14**	**0.44**±**0.07**	**0.31**±**0.19**	**0.25**±**0.08**	**0.11**±0.04*	0.80*

For each seizure, it is presented its EPILEPSIAE ID, the number of seizures used for training and testing and their recording duration, the annotated activity pattern (unclear (?), rhythmic sharp waves (s), rhythmic alpha waves (a), rhythmic delta waves (d), rhythmic theta waves (t), rhythmic beta waves (b), repetitive spiking (r), cessation of inter-ictal activity (c), amplitude depression (m)), seizure classification (unclassified (UC), Focal Onset Aware (FOA), Focal Onset Impaired Awareness (FOIA), Focal to Bilateral Tonic-Clonic (FBTC)), and period of the day at onset (day, between 7 am and 10 pm (D), and night, between 10 pm and 7 am (N)). Training and testing results are also presented, where the last two columns concern the two performance statistical validations (* stands for statistical significance).

It is worth noting the existence of a relation between the number of training seizures and fitness, with a Pearson correlation value of $\rho =-0.49$. We assume this as natural, as it is considered a more difficult task to identify all pre-ictal samples without raising false alarms for a higher number of seizures. Furthermore, relations concerning fitness and testing $S_{p}$ ($\rho =0.19$) and testing FPR/h ($\rho =-0.25$) were also found. These findings may lead us to believe in the existence of concepts drift that can not be handled by simply using in the EA all available data and by retraining the used features with upcoming data. Perhaps, results would differ if a new EA would be executed whenever a new seizure was available, using only the last *N* seizures. In other words, this would require us to re-select our features periodically with the availability of new seizures. Despite this iterative procedure would largely increase computational complexity in our study, along with the necessity of more testing seizures, it could be applied in real life, as our training stage is relatively fast (an EA execution that uses 3 seizures for training and that reaches the maximum number of iterations, takes approximately 2 h 50 min to run on a computer with an Intel Core i7-8700 CPU 3.20GHz 3.19 GHz processor, 32Gb of RAM, on Windows 10 Pro, with Python 3.7 on Spyder 3.3.4). In this study, we tried to take into account computational complexity, as real-time applicability, power-efficiency and minimal computation are important for a real-life context^11^. This is the major reason why we only used the last 4 h of data before each training seizure, as using all available one would enlarge significantly training duration. This is a limitation of this methodology since it is advised to use all inter-ictal control data, as a restriction could introduce a confounding bias^16^.

The performance results from the proposed approach and other studies^17–19^, based on patients from the EPILEPSIAE database, can be compared here. Authors in those studies used lower SPH intervals. As these methods can be used for not only the development of closed-loop systems integrated with seizure-suppression strategies but also used for a patient warning-system only, we opted for a longer SPH in order to account all possibilities. We believe that a 10-min SPH may be a reasonable limit for a patient to minimize seizure consequences, as safely stop driving a car before a seizure. The results reported by Alvarado Rojas et al.^19^ for patients with temporal-focus seizures, despite outperforming the proposed methodology’s sensitivity ($S_{p}= 0.66$), and presenting a lower FPR/h ($FPR/h=0.33$), obtained a percentage of patients performing above chance level of about 10% (3 out of 34), which is lower than ours for any of the three pre-ictal periods. Direito et al.^17^ performed one of the largest studies with EPILEPSIAE concerning the number of patients: 218 with only 11% patient-models performing above chance, and an overall sensitivity of 0.39, which is similar to ours. Nevertheless, it is important to stress that these authors used the random predictor^43^ for statistical validation while we used a surrogate method.

Other studies^18,20,44^ using EPILEPSIAE database could be compared, but in these, several models were trained and tested, where the best predictor was selected based on testing performance. This selection procedure results in an over-estimation of the real performance, given that if a higher number of predictors are tested, the chance to hit seizures, only by chance, increases. This can explain the higher performance obtained. It is somehow limited in real-life application, as it is not possible to choose the best model based on testing values. A fair comparison with the present approach would correspond to select, for each patient, the set of features and corresponding pre-ictal period that best performed on our testing set.

Alvarado Rojas et al.^19^ used a threshold classifier. Concerning features, despite easy to be understood in terms of feature engineering, it may be somehow difficult for a physician to understand the interactions between the phase of low-frequency rhythms (slow waves and theta) and the amplitude of different sub-bands of gamma rhythms. The remaining studies^17,18,20,44^ were based on Support Vector Machines (SVMs) classifiers being fed with the same features as the ones in this work, and with additional ones which can be more difficult to explain to a physician, such as the autoregressive modelling predictive error, decorrelation time, Hjorth parameters, third and fourth-order statistics and energy wavelet coefficients. Additionally, their number of features per model 10 features in average^20^. Based on the above, we believe our methodology, despite not being fully explainable to a physician, it is more intuitive than the remaining. Concerning classifiers, a threshold classifier is clearly the most simple and intuitive, which was the reason behind the usage of a logistic regression: its binary decision also concerns a threshold. Furthermore and as mentioned, it was also inspired by the concept of being more appropriate to consider the seizure prediction as a regression problem^10^, despite its final transformation into a classifier.

These findings lead us to believe that it may be possible to build more interpretable models that perform above chance level for a higher ratio of patients ($\approx$ 32%) when comparing with other more complex methods. However, our methodology is clearly outperformed in terms of FPR/h and sensitivity, which addresses its incapacity to handle the remaining cases and enhances the demand for increasing the model complexity. Additionally, we would like to highlight the existing limitations on these comparisons: the mentioned studies (except for^44^) presented a higher number of patients and seizures, as well as larger inter-ictal periods when comparing to our study. As we had a small number of testing seizures for each patient, our results also present significantly large standard deviations, which must be considered. This was also the reason why we implemented the surrogate predictor, as it is more flexible^16,42^ than the random predictor, it allows for adaptation to the used data and may provide a more solid validation. Moreover, as we are working with data that we have available for unlimited testing, the sliding-step size, number of used features, and number of lag features were reasonably chosen based on computation time, without any tuning concerning testing results. Despite our results may be low in terms of sensitivity and FPR/h, we are avoiding a publication bias by excessively testing our data, as this may constitute a severe problem in this type of data^11^.

Concerning a real-life context, the development of a closed-loop system that disarms seizures by electrical stimulation, which requires iEEG data, seems to be the most viable option, concerning recent developments^10,11,16,45^. Nevertheless, scalp EEG has also some advantages over the iEEG, as it is non-invasive and it would allow for a wider use: it contains fewer risks for the patient and a simple warning system could be cheaper. Another reason that led us to choose the scalp EEG was our objective of providing more knowledge concerning the ictogenesis process along with the network theory, as it proposes that even focal seizures may arise from abnormal activity resulting from a large-scale functional network that spans across lobes and hemispheres^11,16^. Furthermore, our idea of iteratively re-selecting our features by running our EA periodically would also consider the existing dynamics of the epileptic network, which is not static and would provide a greater insight^11^.

**Figure 6:**
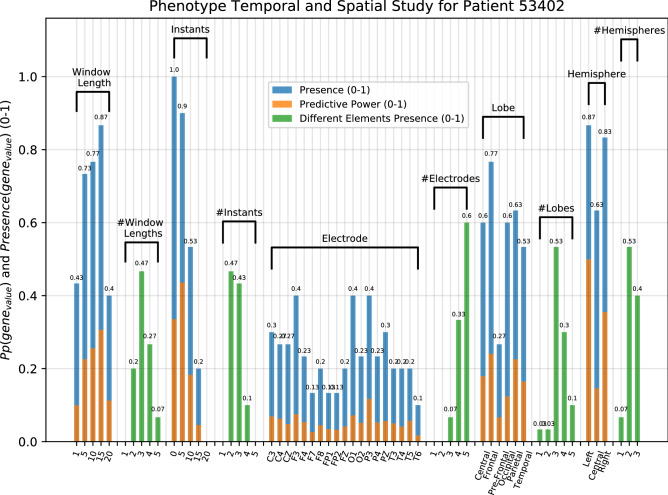
Phenotype temporal and spatial study for patient 53402. The presence and predictive power of temporal (window length and time instant) and spatial (electrode, lobe, and hemisphere) gene values are presented in blue and orange, respectively. The simultaneous presence of different gene values is presented in green.

One of the major reasons to propose this method is the possibility to develop auditable predictors and to extract patient-specific clinical knowledge. Thus, patient 53402 (for a 40-min minimum pre-ictal period) was selected as an example, since the testing results were considered satisfactory ($S_{p}=0.78\pm 0.31$ and FPR/h=$0.35\pm 0.11$, and outperformed the surrogate predictor). Figure 6 presents the phenotype study for the presence ($Presence(gene_{value}$) and predictive power ($Pp(gene_{value}$) of all genes (electrode, window length and time instant genes) from the correspondent obtained hyper-features. Regarding electrodes and their correspondent lobe and hemisphere (spatial study), there are interesting findings related to patient comfort and signal acquisition factors. For instance, it is possible, for this patient, to choose a setting of electrodes that are placed in only three different lobes. In fact, 53% of cases account for electrode placement in three different lobes, which was the most frequent scenario. This can be medically important to understand brain phenomena and to overcome real-life obstacles concerning EEG acquisition and patient comfort.

Looking at the results from the time-related genes (window length and time instant), it is possible to understand the demand for (i) the investigation of simultaneous temporal scales (different window lengths) and (ii) the search for a sequence of events. When considering all the 30 executions, not in just a single one a set of hyper-features used the same window length or used only one single instant. It is possible to see the presence of at least two different time windows and two different time instants for all executions. The most frequent case was the simultaneous presence of two different instants (47% of times). This demonstrates that the EA tends to choose a sequence of two instants. Thus, it searches for a seizure-related pattern instead of searching for a particular feature in a determined instant. Concerning window lengths, at least three different values were present in, at least, 80% of times, where the 1-min window was always present, followed by the 5-min one (90% of times). It is also important to mention that the temporal lobe electrodes were not more chosen than others. Nevertheless, we believe it would be likely that, for a lower SPH, we would have found a significantly higher predictive power in the temporal lobe electrodes, as we would be closer to seizure onset.

Concerning features and mathematical operators, theta band relative power (97% of cases), mean normalized frequency (67% of cases) and medium temporal intensity (60%), and average power (53% of cases) were the most extracted features. For each set of hyper-features, the minimal number of different features was three, where four was the most frequent scenario (60% of times). In all cases, the set of hyper-features presented features from both groups: non-band waves and band-waves (see Supplementary Material for a figure concerning the phenotype study of the decoded features and mathematical operators).

**Figure 7:**
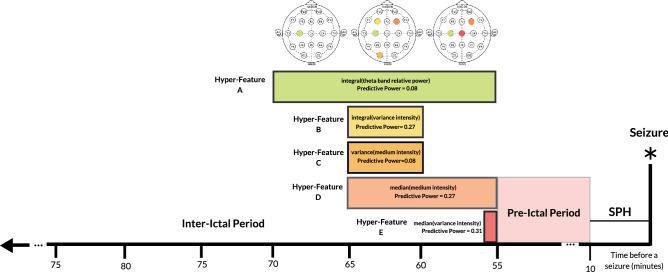
An example of the EA output, for patient 53402, with a pre-ictal period of 55 min, 10-min SPH and a 45-min SOP, where each hyper-feature has a different colour. One can see the sequence of hyper-features: their window length, electrode, feature, and applied mathematical operator.

From the 30 sets of hyper-features, one was selected to demonstrate its interpretability. Thus, the chosen one is represented in Fig. 7 and has a SOP period of 45 min (pre-ictal period of 55 min). Its training performance was $S_{p}$=1.00 and FPR/h=0.00 for training and $S_{p}$=1.00 and FPR/h=0.16 for testing seizures. As it is possible to see, two different feature instants (sequence instants) with four different window lengths are present, as well as a change on the selected electrodes over time, which supports the network theory. This is a model that can be more easily explained to a physician, as the extracted features are relatively simple and in a reduced number, and the used classifier is a logistic regression, instead of a black-box model.

In short, this method offers the possibility of extracting medical knowledge from a different perspective due to the phenotype study. Additionally, it is also possible to find a set of different solutions to find which ones need to be always present and others that have interaction, i.e., which features always appear in the presence of others (association learning). The use of a reduced set of electrodes may provide more comfort to the patient while its acquisition may be more simple. In the case of genes with a significant predictive power when compared to others, it may indicate the existence of key properties for the EEG seizure prediction context.

## Conclusion

This work can be considered as a proof-of-concept study of using EA for seizure prediction. Performance above chance level was achieved for a significant number of patients ($\approx 32\%$) while maintaining interpretability, by accounting synergy between features and all pipeline stages. Despite only 32% patient models have performed above chance level in all three pre-ictal periods, it was possible to develop for 89% of patients a number of executions that were statistically significant for all tested pre-ictal periods, which gives us hope in this methodology. Even though the training stage of this methodology may be computationally expensive and therefore, only the last recorded 4 h before each seizure were used, its real-time application is light and simple: light pre-processing and feature extraction processes, followed by the application of a logistic regression. Nevertheless, our methodology in terms of FPR/h and sensitivity is considerably outperformed by other methodologies using data from the same database, which may indicate the need for higher complexity models.

Despite the obtained percentage of patients performing, an FPR/H<0.15 was not obtained^7^. Moreover, since we used data from surgical monitoring, this study can only be envisioned as a hypothesis. Towards a clinical validation, additional studies must be performed with long-term recordings from patients in their daily life, as the study carried out by Cook et al.^45^. It is also important to mention that we did not test this framework in other types of epilepsies, which concerns future work. We plan on testing the robustness of this approach in patients with other types of epilepsy including both focal onset (e.g., frontal lobe epilepsy) and generalized onset. We believe these results can be improved and that this methodology, combined with other developed approaches, confounding variables and other biosignals^10–12,16,41^, can help the design of novel prediction algorithms aiming at clinical acceptance.

## Supplementary Information

Supplementary Information
