# Supplementary material for: A personalized and evolutionary algorithm for interpretable EEG epilepsy seizure prediction
Source: Sci Rep. 2021 Feb 9;11:3415. doi: 10.1038/s41598-021-82828-7 (PMC7873127; doi:10.1038/s41598-021-82828-7)
Supplement: Supplementary file 1 — Supplementary Information [file 41598_2021_82828_MOESM1_ESM.pdf]

A Personalized and Evolutionary Algorithm for Interpretable EEG Epilepsy Seizure Prediction

Mauro. F. Pinto[1,*], Adriana Leal[1], Fábio Lopes[1], António Dourado[1], Pedro Martins[1], and César A. Teixeira[1]

[1]Univ Coimbra, CISUC-Center for Informatics and Systems of the University of Coimbra, Department of Informatics Engineering, Coimbra, Portugal
[*]mauropinto@dei.uc.pt

Supplementary Material

Evolutionary Algorithm Configuration Details

In parent selection, a binary tournament was used. The population was shuffled and all paired consecutive individuals competed, e.g.: individual #1 competed with individual #2, individual #2 competed with individual #3, ... individual #(N-1) competed with individual #N and individual #N competed with individual #1. By performing the tournament in these terms, the best individual is always selected twice and selective pressure is controlled.

Regarding variation operators, we used recombination followed by mutation. After selecting our parents, this mating pool was shuffled and a similar tournament approach was used to guarantee that the best individual would enter in four recombination operations: individual #1 recombined with individual #2, individual #2 recombined with individual #3, ... individual #(N-1) recombined with individual #N and individual #N recombined with individual #1. Recombination was performed with a given probability (80%): when a parents pair did not recombine, one of them survived and was passed to the offspring population, after mutation. In each generation, we created an offspring population from the same size as the one from the current population (100 individuals).

Genotype-Phenotype Mapping Example

We provide here a Genotype-Phenotype mapping example. Suppose that for a 10-minute SPH and for a minimum pre-ictal period of 30 minutes, a given individual is represented by three (not five for simplicity reasons) hyper-features, whose codification is presented in Table S1.

The genotype-phenotype mapping transforms the genotype of an individual in a set of hyper-features extractor that can perform sliding time-window analysis. The first three decoding steps consist in: i) finding the features that will be decoded to the phenotype; ii) constructing the hyper-features using the decoded features and the remaining genes; and iii) placing the hyper-features chronologically and obtaining the pre-ictal time.

Concerning step i), for hyper-features A and B, the decoded features are the alpha and beta band-waves, respectively since their dominant feature gene is the band-wave one. As the dominant feature gene in C is the non-band wave one, the decoded feature is the average power. This step is shown in S1, more specifically in red.

Concerning step ii), the selected features, alpha band, beta band and average power, will now be collected for constructing hyper-features A, B, and C from electrodes Cz, O1 and T6, in windows of 15, 5 and 1 minutes, respectively. All possible window lengths are multiples of 5 seconds as the features were beforehand extracted in windows of 5 seconds, as described in the Pre-Processing and Feature Extraction section. Then, to these windows, the correspondent mathematical operator is applied: the variance, the mean and the integral for hyper-features A, B, and C respectively.

Table S 1. Example of an individual genotype. In this example, an individual is composed by only three hyper-features and not five, due to simplicity reasons. Each hyper-feature comprises seven genes: dominant feature, band-wave feature, non-band wave feature, mathematical operator, electrode, window length and time instant.

	Dominant Feature	Band Wave Feature	Non-Band Wave Feature	Mathematical Operator	Electrode	Window Length	Time Instant
**Hyper-feature A**	Band wave	Alpha	Average Power	Variance	Cz	15 minutes	30 minutes
**Hyper-feature B**	Band wave	Beta	Mean. Norm. Freq.	Mean	O1	5 minutes	10 minutes
**Hyper-feature C**	Non-Band wave	Beta	Average Power	Integral	T6	1 minute	20 minutes

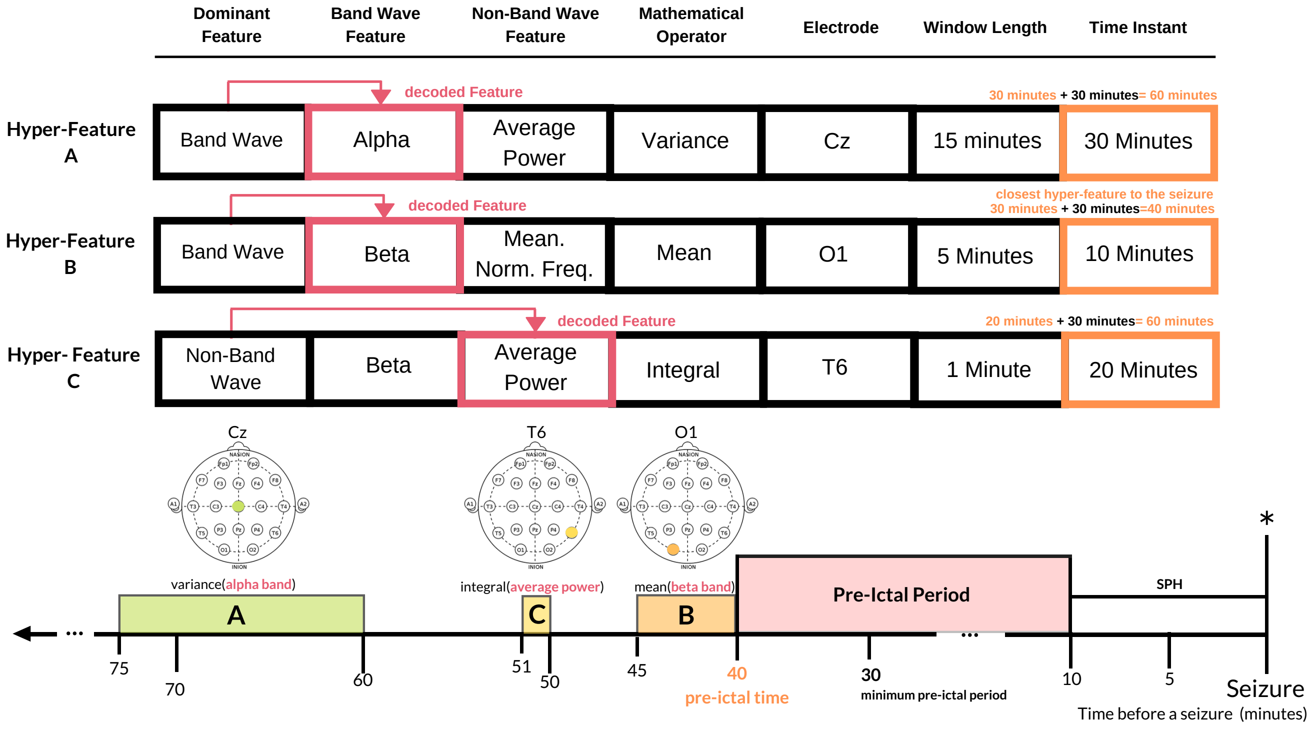

S 1. The first three steps of decoding the genotype into the phenotype: i) decoding the dominant feature (in red); ii) constructing the hyper-features using the decoded feature and the remaining genes (in the timeline); iii) determining the hyper-features temporal position and finding the pre-ictal period (in orange).

Concerning step iii), each hyper-feature temporal position is obtained by adding to the minimum pre-ictal period the correspondent time instant. This gene allows analysing a sequence of instants instead of only one instant. Additionally, it also allows adapting the pre-ictal period duration, as it is determined by calculating the temporal distance from the first chronological hyper-feature to seizure onset. For a better understanding, this step is demonstrated in S1, more specifically in orange.

Then, by setting the decoded hyper-features chronologically concerning the used pre-ictal period, it is possible to perform label (pre-ictal/inter-ictal) and hyper-feature extraction through sliding-window analysis, both in training and testing seizures for fitness function evaluation, as depicted in S2.

Neighbourhood Details

We provide here more details concerning the established neighbourhoods. Window length and time instant genes have a straightforward ordering, as their values correspond to increasing/decreasing discrete time intervals. Electrodes' neighbourhood is based on their scalp position. As there is no decreasing/increasing relationship among the mathematical operators (mean, median, variance, integral), all genes were considered neighbours of each other. The extracted features, however, can be divided into band waves and not-band waves. While the first can be ordered by their frequency range (e.g., delta, theta, alpha, beta, gamma), mean normalized frequency, average power, variance or mean intensity do not have an apparent order. Thus, it was necessary to split these into two groups and to create the dominant feature gene to decide which feature is decoded. Consequently, each hyper-feature has information in its genotype for a band-wave feature and for a non-band wave feature, but only one is decoded, as depicted in Fig. 3.b).

Mutation Operator Details and Example

Mutation, interpreted as a unitary step that will cause a random and unbiased change[1], occurs in the following form for an individual: one of the hyper-features is chosen randomly, and then one gene of that hyper-feature is chosen randomly to mutate. The remaining hyper-features and genes continue unaltered. The gene selection, despite random, is made by considering a weighted-probability according to its neighbourhood: a gene's probability to be selected is proportional to its number of possible neighbourhood values. The higher the number of possible values (number of neighbors) for a gene, the higher its probability to be selected for mutation. Thus, the gene i probability selection $g(i)$ is computed as in (1), where G is the number

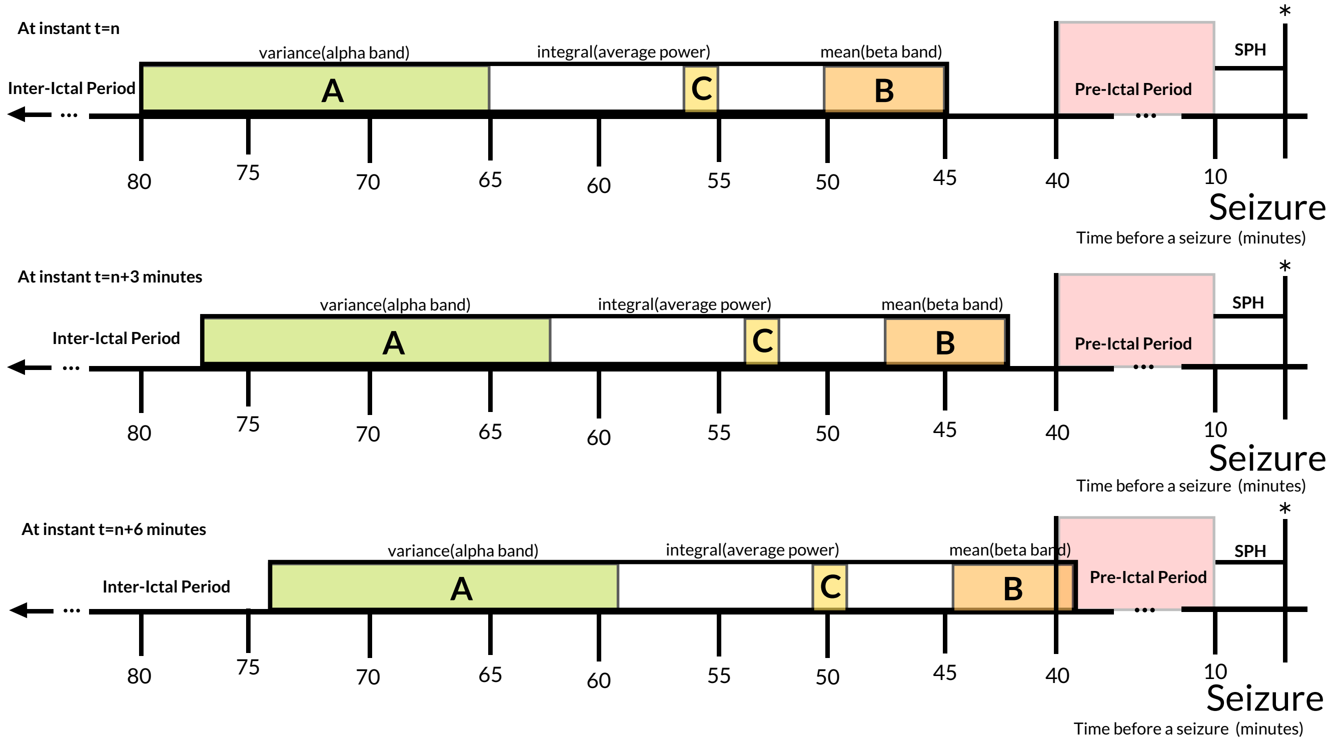

S 2. With the hyper-features ordered chronologically, it is possible to perform feature extraction for a time-moving analysis and to label instants as belonging to the pre-ictal/inter-ictal period.

of genes and $N_N(i)$ the gene respective number of neighbors:

$$g(i) = \frac{N_N(i)}{\sum_{j=1}^{G} N_N(j)}. \tag{1}$$

To perform a unary step, depending on the gene and its value, the mutation operator will act differently since different genes have different neighbourhoods. One can understand all gene neighbourhoods as graphs: time instants, window-length and wave feature domains are graphs where the connected nodes have ordered values. Mathematical operator and non-wave features are graphs where each node is connected to all nodes. Thus, a mutation can be interpreted as a random change from a gene node to a neighbouring one, randomly chosen.

We provide here an example using the same individual from Table S1. The mutation operator occurs in the following form, as illustrated in S3: one of the hyper-features that composes the individual is chosen randomly (hyper-feature C), and then one gene of that hyper-feature is chosen randomly to mutate (electrode gene). Mutation will perform a random change from the current gene value node (T6) to one of its neighbours (T4, P4, C4, O2), in this case, to T4 gene value. The remaining hyper-features and remaining genes from the mutated hyper-feature remain the same.

Recombination Operator Details and Example

Recombination is a stochastic operator that combines genetic information from two parents (individuals) into one or more offspring[1]. After selecting two parents to reproduce, this operator performs the recombination of all paired hyper-features. Thus, hyper-feature pairing is the first step and is performed by calculating their Manhattan distances and matching the closest ones. The distance between two hyper-features is equal to the number of steps needed to go from one value to another by taking the shortest path. By representing $D(f_a, f_i)$ as the distance between the hyper-features f_a and f_i where a is the index of the fixed feature from one parent and i the feature index that iterates the other parent F number of features, the matched feature index m will be given by equation (2). Thus, features f_m and f_a match.

$$m = \arg\min_i D(f_a, f_i), 1 \leq i \leq F \tag{2}$$

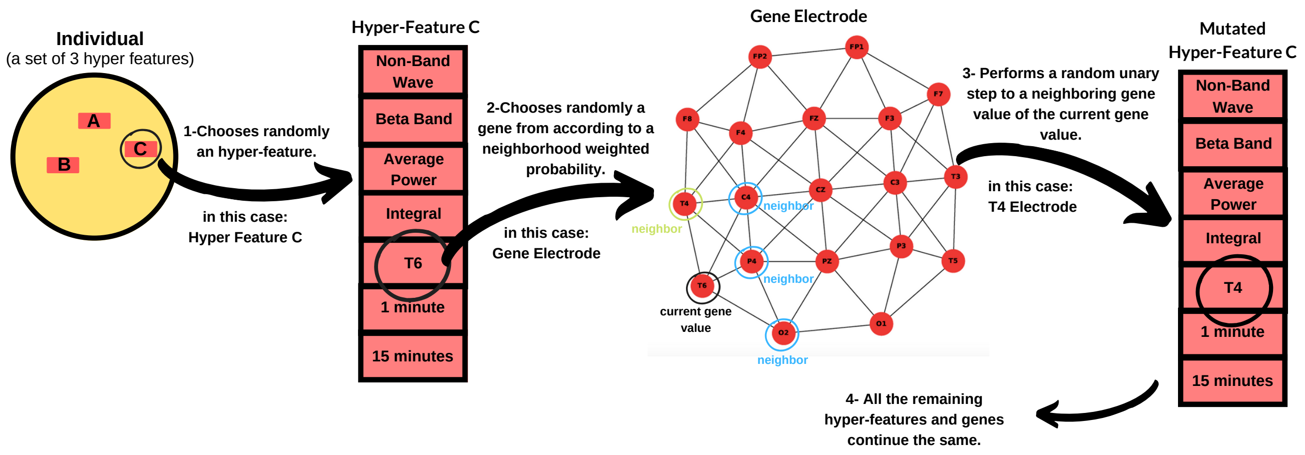

S 3. How the mutation operator works in one individual.

$D(f_a, f_i)$ can be described as the summation of all gene Manhattan distances, where G is the number of genes from an feature and $d(f_{a_i}, f_{b_i})$ is the distance between two i gene values from features f_a and f_b, presented in equation (3).

$$D(f_a, f_b) = \sum_{i=1}^{G} d(f_{a_i}, f_{b_i}), 1 \leq i \leq G \tag{3}$$

After the hyper-feature matching operation is finished, the recombination operator works at the hyper-feature gene level. Consequently, each offspring gene value was obtained by choosing a random node belonging to the shortest path between the correspondent two parent gene values. The distance between two gene values is equal to the number of steps needed to go from one value to another by taking the shortest path. Due to computational simplifications, the feature matching step did not take into account the total distance of the minimum components as it used a greedy approach. More specifically, one of the parents was selected and for each feature in his genotype, the closest feature from the other parent was matched. This was done iteratively which means that if a feature was already matched with a previous component, it could not be used, even if a lower $D(f_a, f_b)$ was obtained with the new matching.

Concerning recombination at the hyper-feature gene level, we provide here an example between two hyper-features. Thus, in this stage, hyper-features from both parents were already paired. S4 illustrates the recombination operation concerning one of the paired hyper-features (orange and red), where each gene is recombined (the same process is then repeated with all paired hyper-features). The new gene value is a random node between the shortest path of the two parent gene nodes. When several possible paths are possible, as the case of the electrodes, one of the shortest paths is, beforehand, randomly chosen. The recombined hyper-feature is presented in green.

Fitness Function Details

Concerning the logistic regression classifier training, a balanced weighting was applied to adjust the class weights to become inversely proportional to their frequency of occurrence. The weight of class i, C_{w_i}, is given by Eq. (4), where N_S is the total number of training samples, N_C is the number of classes (pre-ictal and inter-ictal) and N_{C_i} is the number of samples from class C_i. With this, samples belonging to the pre-ictal period, that are less frequent, will have a higher weight.

$$C_{w_i} = \frac{N_S}{N_C N_{C_i}}. \tag{4}$$

With regard to the Firing Power[2] technique, it consists in a moving average filter that can be interpreted as a voting decision filter described in Eq. (5) where $O(t)$ is the output of the trained logistic regression at instant t and F_P the filter-size which corresponds to the pre-ictal period duration:

$$FiringPower(t) = \begin{cases} alarm & \text{if } \sum_{x=0}^{x=F_P-1} \frac{O(t-x)}{F_P} \geq threshold \\ no\ alarm & \text{if } \sum_{x=0}^{x=F_P-1} \frac{O(t-x)}{F_P} < threshold \end{cases}. \tag{5}$$

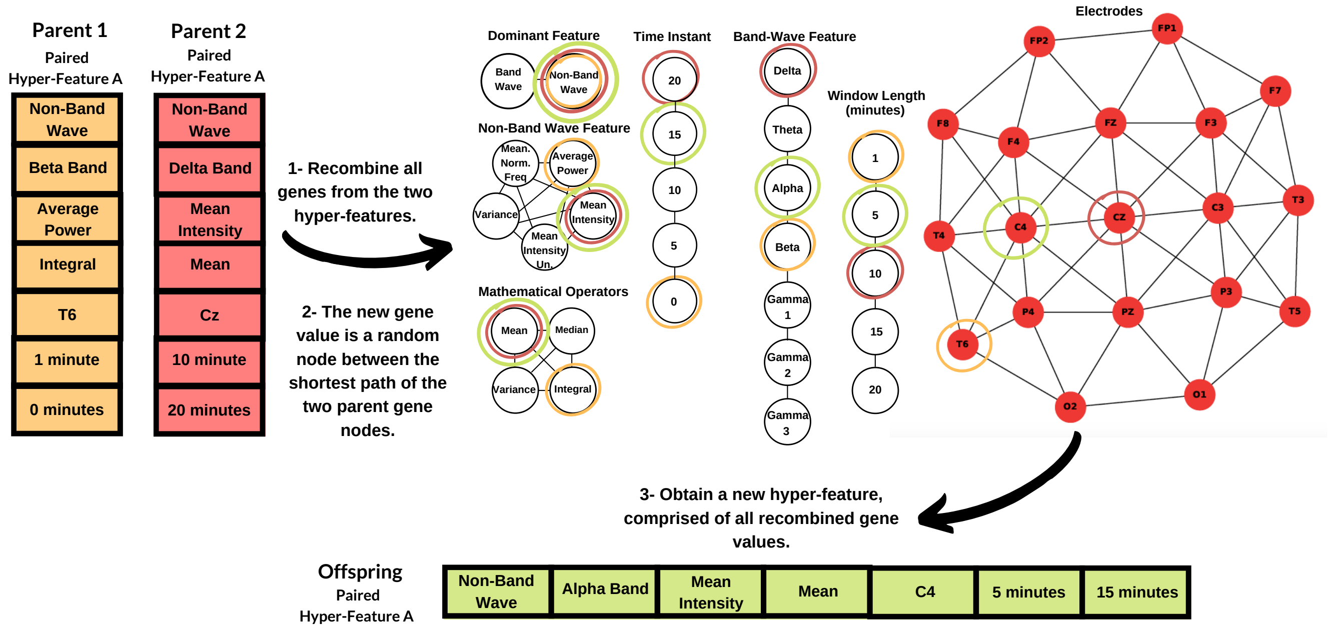

S 4. An example of how the recombination operator work at the hyper-feature gene level. The genotype of the parents' hyper-features is presented in orange and red, while green represents the recombined hyper-feature.

The threshold value can be seen as the maximum tolerance for the predictor error. Above this threshold, an alarm is fired. The threshold was set to a reasonable limit of 0.70. The idea of using a defined threshold is to admit noise and, in this case with post-processing, to reduce the probabilities of the EA to overfit the training samples. With a higher threshold, a more conservative system is obtained.

Surrogate Analysis Details

The surrogate predictor makes use of Monte Carlo simulations by random shifting seizure times. Thus, for each tested seizure for each execution, the correspondent seizure times were randomly shifted 30 times and the surrogate performance was compared with the predictor one. A model is considered to perform above chance if its performance is higher than the surrogate one with statistical significance, under the following null hypothesis that the proposed method performance is not above chance level.

Moreover, by shifting the seizure times for each tested seizure separately, we accounted for the non-random occurrence of seizures. For example: if one seizure has 10 hours of recorded data and another has 30 hours, it would not be rigorous to simply generate two random seizure times within the entire recording of 40 hours. In short, we randomly shift the seizure time of the first seizure within its correspondent 10 hours, and the other seizure time within its correspondent 30 hours. Concerning a possible diurnal variation in seizure distribution, we decided to not take it into account, as our training data only contained the last 4 recorded hours before each seizure and since our models are unspecific for confounding factors.

Phenotype Study Mathematical Formulation

As EAs are associated with random components (in this case: initialization, parent selection, and evolution operators), it is possible to obtain, for each execution, a different solution (set of hyper-features) with similar performance. Thus, the objective of performing a phenotype study is to understand the overall influence of each gene value concerning the obtained hyper-features.

For a given hyper-feature j, a simple approach can study each decoded gene individually. It is possible to calculate the gene value predictive power $pp(gene_i, value)$ from a gene $gene_i$ using Eq. (6), by assigning to it the absolute of the correspondent logistic regression coefficient. Presence was also studied, where a binary value (1/0) was assigned considering the gene value

presence in a hyper-feature (7).

$$pp(gene, value) = \begin{cases} abs(\beta_j), & \text{if } gene = value. \\ 0, & \text{otherwise.} \end{cases}, \tag{6}$$

$$presence(gene, value) = \begin{cases} 1, & \text{if } gene = value. \\ 0, & \text{otherwise.} \end{cases}. \tag{7}$$

Then, by applying the previous equation for all F hyper-features that compose an individual, one obtains the correspondent gene value predictive power for an individual. After this, one can compute the correspondent normalized gene value predictive power $Pp(value)$ and normalized presence $Presence(value)$ for all individuals I, as demonstrated in Eq. (8) and (9), respectively. As 30 executions were performed and as each individual is composed of 5 hyper-features, $I = 30$, $F = 5$, and G gene values (which depends on the gene):

$$Pp(value) = \frac{\sum_{i=1}^{I}\sum_{j=1}^{F}\sum_{k=1}^{G} pp(gene_{ijk}, value)}{\sum_{i=1}^{I}\sum_{j=1}^{F}\sum_{k=1}^{G} abs(\beta_j)}, \tag{8}$$

$$Presence(value) = \frac{1}{I}\sum_{i=1}^{I} \max\left\{presence(gene_{ijk}, value) | j = 1, \ldots, F; k = 1, \ldots, G\right\}. \tag{9}$$

Results - All patient-models

Table S2 presents the performance results for all patients, for all minimum pre-ictal periods.

	Training		Testing					
Patient ID	SOP (minutes)	Fitness	S_p	FPR/h	Surrogate S_p	Above Chance	Ratio Executions Above Chance	Significance for whole set
11002	33.67±3.64	0.64±0.06	0.27±0.31	1.57±2.58	0.34±0.18		0.17	*
	43.17±3.02	0.67±0.11	0.07±0.21	0.57±0.29	0.22±0.13		0.10	*
	52.83±3.34	0.82±0.09	0.12±0.21	0.37±0.15	0.25±0.14		0.17	*
16202	31.00±2.00	0.58±0.09	0.30±0.26	0.49±0.33	0.16±0.09	*	0.60	*
	43.83±3.80	0.57±0.10	0.12±0.22	0.40±0.24	0.16±0.09		0.27	*
	52.83±3.58	0.75±0.09	0.13±0.18	0.39±0.20	0.21±0.09		0.33	*
30802	36.33±4.27	0.36±0.10	0.58±0.35	3.77±8.02	0.39±0.16	*	0.50	*
	43.17±3.02	0.71±0.11	0.44±0.28	0.63±0.64	0.33±0.14		0.63	*
	50.83±2.61	0.46±0.08	0.70±0.20	0.55±0.47	0.40±0.12	*	0.90	*
**53402**	**34.33±3.82**	**0.76±0.11**	**0.63±0.41**	**0.50±0.35**	**0.22±0.06**	*****	**0.77**	*****
	**43.83±2.79**	**0.84±0.08**	**0.78±0.31**	**0.35±0.11**	**0.22±0.08**	*****	**0.93**	*****
	**51.67±2.69**	**0.92±0.05**	**0.67±0.30**	**0.34±0.13**	**0.24±0.08**	*****	**0.90**	*****
**55202**	**31.83±2.41**	**0.59±0.04**	**0.70±0.22**	**1.21±0.42**	**0.36±0.09**	*****	**0.90**	*****
	**43.67±3.86**	**0.63±0.07**	**0.67±0.27**	**1.03±2.23**	**0.35±0.11**	*****	**0.67**	*****
	**53.00±3.56**	**0.72±0.06**	**0.70±0.23**	**0.53±0.28**	**0.32±0.13**	*****	**0.87**	*****
58602	30.50±1.50	0.59±0.14	0.38±0.25	2.14±0.98	0.45±0.15		0.33	*
	42.50±3.10	0.38±0.21	0.17±0.19	0.53±0.52	0.21±0.17		0.33	*
	51.67±2.69	0.48±0.11	0.16±0.17	0.25±0.27	0.12±0.11		0.33	*
60002	35.00±4.08	0.68±0.12	0.48±0.30	1.29±0.52	0.36±0.09		0.60	*
	43.33±3.73	0.71±0.08	0.43±0.36	1.18±0.48	0.39±0.09		0.47	*
	52.83±2.79	0.85±0.05	0.37±0.22	1.06±0.35	0.39±0.10		0.33	*
64702	35.17±3.53	0.55±0.23	0.02±0.09	0.92±0.45	0.20±0.07		0.00	
	44.17±3.67	0.71±0.14	0.05±0.15	0.73±0.39	0.18±0.05		0.10	*
	53.50±2.29	0.68±0.24	0.00±0.00	0.37±0.12	0.14±0.05		0.00	

Table 2 continued from previous page

	**31.67±2.98**	**0.59±0.10**	**0.70±0.22**	**0.69±0.33**	**0.28±0.07**	*	**0.97**	*
**75202**	**43.33±2.98**	**0.62±0.11**	**0.80±0.22**	**1.03±1.60**	**0.31±0.16**	*	**0.87**	*
	**51.67±2.36**	**0.62±0.12**	**0.84±0.22**	**0.93±1.11**	**0.37±0.14**	*	**0.87**	*
	34.50±3.95	0.50±0.08	0.31±0.21	0.65±0.34	0.31±0.10		0.43	*
80702	43.17±2.41	0.63±0.09	0.31±0.27	0.69±0.28	0.41±0.13		0.30	*
	51.33±2.21	0.63±0.13	0.34±0.25	0.80±0.27	0.50±0.11		0.20	*
	**34.67±2.87**	**0.71±0.04**	**0.47±0.18**	**0.36±0.34**	**0.16±0.08**	*	**0.90**	*
**85202**	**40.50±1.50**	**0.69±0.05**	**0.45±0.15**	**0.36±0.19**	**0.17±0.07**	*	**0.90**	*
	**53.67±2.56**	**0.72±0.07**	**0.43±0.21**	**0.35±0.30**	**0.19±0.11**	*	**0.83**	*
	36.17±2.11	0.63±0.06	0.38±0.24	1.05±0.58	0.31±0.13		0.53	*
94402	41.67±2.69	0.60±0.10	0.27±0.29	0.85±0.83	0.31±0.17		0.33	*
	50.83±1.86	0.64±0.04	0.23±0.26	0.82±0.66	0.35±0.08		0.17	*
	35.33±3.14	0.52±0.12	0.14±0.17	0.66±0.34	0.23±0.09		0.27	*
95202	44.00±4.16	0.67±0.06	0.00±0.00	0.58±0.23	0.27±0.06		0.00	
	54.67±2.87	0.76±0.07	0.00±0.00	0.45±0.27	0.26±0.07		0.00	
	30.50±1.98	0.52±0.12	0.23±0.20	0.77±0.53	0.24±0.10		0.43	*
96002	42.83±4.22	0.41±0.10	0.31±0.19	1.25±1.80	0.33±0.15		0.40	*
	53.00±3.06	0.62±0.05	0.33±0.17	0.69±0.66	0.30±0.12		0.40	*
	33.50±3.69	0.55±0.17	0.19±0.24	1.72±1.43	0.35±0.13		0.13	*
98202	42.00±2.77	0.54±0.16	0.31±0.32	1.66±1.23	0.35±0.18		0.33	*
	52.17±2.79	0.69±0.09	0.24±0.24	0.76±0.55	0.27±0.16		0.23	*
	33.00±2.77	0.55±0.08	0.23±0.28	0.44±0.28	0.17±0.10		0.43	*
101702	41.67±2.98	0.53±0.14	0.22±0.25	0.86±0.76	0.29±0.18		0.10	*
	51.67±2.69	0.58±0.13	0.15±0.23	0.42±0.36	0.23±0.13		0.17	*
	34.67±2.87	0.67±0.08	0.52±0.38	1.29±0.98	0.46±0.14		0.37	*
109502	44.17±4.10	0.65±0.11	0.68±0.33	1.18±0.88	0.46±0.16	*	0.60	*
	54.17±3.44	0.77±0.16	0.83±0.24	1.35±1.35	0.58±0.12	*	0.67	*
	**33.17±3.98**	**0.80±0.10**	**0.47±0.29**	**0.33±0.12**	**0.15±0.07**	*	**0.80**	*
**110602**	**41.50±2.63**	**0.75±0.16**	**0.45±0.27**	**0.31±0.14**	**0.18±0.08**	*	**0.80**	*
	**52.50±3.59**	**0.79±0.08**	**0.50±0.26**	**0.33±0.24**	**0.21±0.11**	*	**0.80**	*
	**33.67±3.14**	**0.44±0.07**	**0.31±0.19**	**0.25±0.08**	**0.11±0.04**	*	**0.80**	*
**114902**	**42.83±2.79**	**0.38±0.12**	**0.31±0.23**	**0.20±0.06**	**0.12±0.05**	*	**0.73**	*
	**51.17±2.48**	**0.45±0.05**	**0.36±0.27**	**0.18±0.04**	**0.13±0.05**	*	**0.67**	*

Table S 2. All patient-model results.

Patient 53402 - Features' Phenotype Study

S5 depicts the gene presence and predictive power of features and mathematical operators.

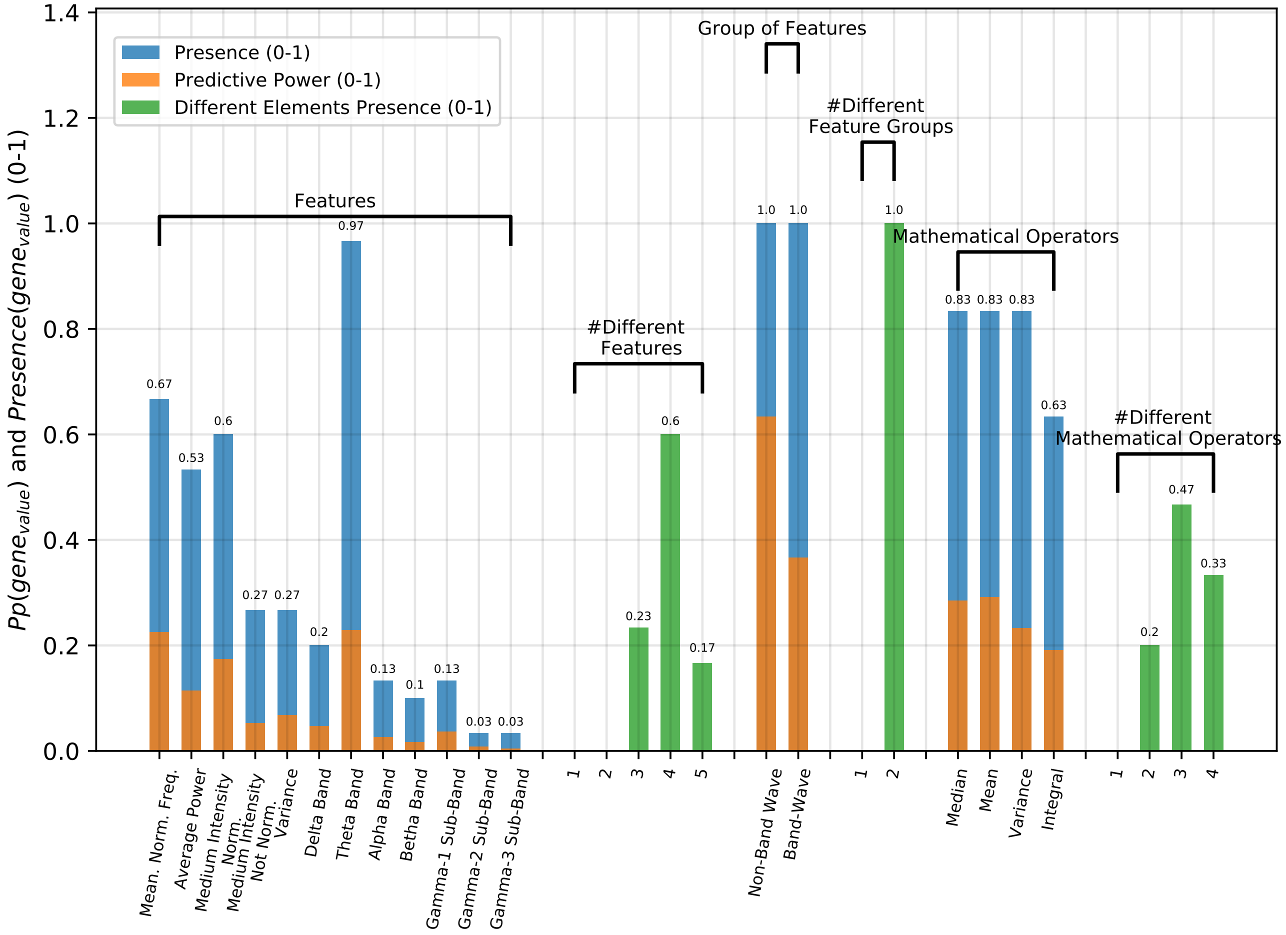

S 5. Genotype features and mathematical operators study for patient 53402, where the presence and predictive power for each feature and operator are presented in blue and orange, respectively. The simultaneous presence of different features and operators in each EA individual are presented in green.

References

1. Eiben, A. E. & Smith, J. E. *What is an Evolutionary Algorithm?*, 15–35 (Springer Berlin Heidelberg, Berlin, Heidelberg, 2003).
2. Teixeira, C. A. *et al.* EPILAB: A Software Package for Studies on the Prediction of Epileptic Seizures. *J. Neurosci. Methods* **200**, 257–271, DOI: 10.1016/j.jneumeth.2011.07.002 (2011).
